# Supplementary material for: GDF15 controls primary cilia morphology and function thereby affecting progenitor proliferation
Source: Life Sci Alliance. 2024 May 7;7(7):e202302384. doi: 10.26508/lsa.202302384 (PMC11077589; doi:10.26508/lsa.202302384)
Supplement: Supplementary file 1 [file LSA-2023-02384_TableS1.docx]

**Supplementary Table S1:** Antibodies used for immunofluorescence. N/A = not available.

| Antigen | Host | Company, Catalog # | Lot # | Concentration |
| --- | --- | --- | --- | --- |
| ADCY3 | rabbit | Thermo Fisher, PA5-35382 | UL2902981 | 1:500 |
| ARL13B | mouse | UC Davis, 75-287 BioLegend, 857602 | 472-1JU-55 B323369 | 1:500 |
| GFRAL | sheep | Invitrogen, PA5-47769 | UH2824346A | 1:200 |
| HDAC6 | rabbit | Proteintech, 12834-1-AP | 00053805 | 1:500 |
| Ki67 | rabbit | Abcam, 16667 | GR3313195-18 | 1:100 |
| Tubulin, acetylated | mouse | Sigma Aldrich, T6793 | 017M4806V | 1:1000 |
| Tubulin, gamma | rabbit | Sigma Aldrich, T5192 | N/A | 1:500 |
