# Supplementary material for: GDF15 controls primary cilia morphology and function thereby affecting progenitor proliferation
Source: Life Sci Alliance. 2024 May 7;7(7):e202302384. doi: 10.26508/lsa.202302384 (PMC11077589; doi:10.26508/lsa.202302384)
Supplement: Supplementary file 2 [file LSA-2023-02384_TableS2.docx]

**Supplementary Table S2:** qPCR probes for TaqMan assays.

| Gene | Assay # |
| --- | --- |
| β-actin (Actb) | Mm00607939_s1 |
| Adcy3 | Mm00460371_m1 |
| Gli1 | Mm00494654_m1 |
| Hdac6 | Mm00515945_m1 |
