# Supplementary material for: GDF15 controls primary cilia morphology and function thereby affecting progenitor proliferation
Source: Life Sci Alliance. 2024 May 7;7(7):e202302384. doi: 10.26508/lsa.202302384 (PMC11077589; doi:10.26508/lsa.202302384)
Supplement: Supplementary file 3 [file LSA-2023-02384_TableS3.docx]

**Supplementary Table S3:** Antibodies used for western blot. *horseradish-peroxidase-conjugated secondary antibody. N/A = not available.

| Antigen | Host | Company, Catalog # | Lot # | Concentration |
| --- | --- | --- | --- | --- |
| Actin | rabbit | Cell Signaling, 4970 | N/A | 1:10000 |
| ERK | rabbit | Cell Signaling, 4695 | 8 | 1:1000 |
| ERK, phospho | rabbit | Cell Signaling, 4370S | 28 | 1:1000 |
| Tubulin, acetylated | mouse | Sigma Aldrich, T6793 | 017M4806V | 1:4000 |
| Tubulin, alpha | mouse | Sigma Aldrich, T9026 | N/A | 1:10000 |
| Mouse* | goat | Jackson ImmunoResearch, 115-035-005 | N/A | 1:10000 |
| Rabbit* | goat | Jackson ImmunoResearch,  111-035-003 | N/A | 1:10000 |
